# Implant Stability of Biological Hydroxyapatites Used in Dentistry

**DOI:** 10.3390/ma10060644

**Published:** 2017-06-12

**Authors:** Maria Piedad Ramírez Fernández, Sergio A. Gehrke, Patricia Mazón, Jose L. Calvo-Guirado, Piedad N. De Aza

**Affiliations:** 1 Cátedra Internacional de Investigación en Odontología, Universidad Católica San Antonio de Murcia, Avda. Jerónimos, 135, 30107 Guadalupe, Spain; jlcalvo@ucam.edu; 2 Biotecnos Research Center, Rua Dr. Bonazo No. 57, Santa Maria 97015-001, Brasil; sergio.gehrke@hotmail.com; 3 Departamento de Materiales, Óptica y Tecnologia Electrónica, Universidad Miguel Hernández, Avda. Universidad s/n, 03202 Elche, Spain; pmazon@umh.es; 4 Instituto de Bioingenieria, Universidad Miguel Hernandez, Avda. Ferrocarril s/n, 03202 Elche, Spain; piedad@umh.es

**Keywords:** hydroxyapatite, xenografts, implant design, implant surface

## Abstract

The aim of the present study was to monitor implant stability after sinus floor elevation with two biomaterials during the first six months of healing by resonance frequency analysis (RFA), and how physico-chemical properties affect the implant stability quotient (ISQ) at the placement and healing sites. Bilateral maxillary sinus augmentation was performed in 10 patients in a split-mouth design using a bobine HA (BBM) as a control and porcine HA (PBM). Six months after sinus lifting, 60 implants were placed in the posterior maxilla. The ISQ was recorded on the day of surgery from RFA at T1 (baseline), T2 (three months), and T3 (six months). Statistically significant differences were found in the ISQ values during the evaluation period. The ISQ (baseline) was 63.8 ± 2.97 for BBM and 62.6 ± 2.11 for PBM. The ISQ (T2) was ~73.5 ± 4.21 and 67 ± 4.99, respectively. The ISQ (T3) was ~74.65 ± 2.93 and 72.9 ± 2.63, respectively. All of the used HAs provide osseointegration and statistical increases in the ISQ at baseline, T2 and T3 (follow-up), respectively. The BBM, sintered at high temperature with high crystallinity and low porosity, presented higher stability, which demonstrates that variations in the physico-chemical properties of a bone substitute material clearly influence implant stability.

## 1. Introduction

The edentulous ridge in the posterior maxilla often presents a limited bone volume due to both a lack of alveolar bone after ridge remodeling and maxillary sinus pneumatization [1]. Adequate alveolar ridges play a crucial role when it comes to rehabilitation with implants, and some augmentation technique is necessary for patients who suffer from alveolar atrophy [2]. The most predictable and commonly used means of facilitating implant therapy in the atrophic posterior maxilla has been sinus augmentation [3]. Although alternatives, e.g., using shorter implants, are beginning to be investigated [4,5,6], any available scientific evidence is modest and insufficient to conclude that the success of sinus lift procedures in bone with a residual height between 4 mm and 9 mm will be better, or not, than when short implants are used [7,8]. Maxillary sinus floor grafting has become the most common surgical intervention when increasing alveolar bone height before placing endosseous dental implants in the posterior maxilla [9]. 

Several factors influence maxillary sinus floor grafting results: specific surgical techniques, a simultaneous versus a delayed procedure, using barrier membranes over the lateral window, implant surface characteristics, the length and width of implants, and selecting the graft material [10]. 

As regards the last factor/variable, researchers have not reached an agreement about the most suitable material for sinus augmentation [11]. In the clinical practice, the main purpose of bone augmentation procedures is bone formation, where implants are positioned to best support prosthetic rehabilitation. The bone tissue around dental implants must be mechanically competent after augmentation procedures [12]. 

Today not many controlled research works evaluate the use of different bone grafting materials for sinus augmentation. Sinus elevation, performed with a wide variety of different graft materials, has been used. However, it still remains unclear which is the most suitable bone grafting material for enhancing bone regeneration in the augmented sinus [13,14]. Deproteinized xenografts, constituted primarily of natural apatites, which are either sintered or not, have good physical and physico-chemical properties. Deproteinization is an indispensable process followed to eliminate antigenicity in xenograft bones. Different physicochemical conditions selectively modulate the host organism’s tissue response [15]. Some authors have reported the same observations in reaction to the microscopic structure in commercial products subjected to thermal deproteinization processes [16,17]. The sintering temperature is considered an important factor that might alter the HA´s characteristics [18]. However, the sintering temperature effect on the physico-chemical properties of natural HA (HA of a natural source), especially HA from bovine bone, is still not fully understood and research in this area is still wide open.

The ultra-structural interface of the graft bone tissue interface, as well as the ideal time point of placing implants, have not yet been described [19].

Typical surgical protocols are simultaneous one-stage lateral or crestal antrostomy when the residual crestal bone is greater than 3–6 mm, or a two-stage delayed procedure, which is recommended when the residual bone is less than 3–6 mm. Furthermore, the risk of implant failure is halved when the two-stage technique is used [20]. 

The graft consolidation gradient reflects the features of each bone substitute at sinus augmentation sites [21]. According to some studies, the physico-chemical properties of each bone material graft may influence the osteointegration process, and this influence may result in shorter healing times between implant placement and restoration. Therefore, a profound understanding of not only the different aspects of biomaterial properties, but also of their relation to and influence on bone healing has proved to be of utmost importance [22].

An undisturbed healing period of at least 3–6 months at surgical sites is the generally accepted protocol after implant placement. This can ensure uneventful healing and improve osseointegration between the implant and bone. The reason for this approach is based on the fact that the functional force around the bone-implant interface causes implant micromotion during wound healing, while implant micromotion may induce fibrous tissue rather than bone contact, which results in clinical failure. All of these concerns about waiting periods have long since been a challenge for both patients and clinicians. Changing trends and demands have rendered the introduction of early loading techniques necessary as a result of searching for faster dental function restoration using implants [23]. Successful osseointegration is a prerequisite for functional dental implants; absence of osseointegration has been reported for implants with no primary stability [24]. 

Implant stability can be defined as the combination of both mechanical and biological stability. While mechanical stability appears as the result of bone tissue compression during implantation, biological stability is obtained as a result of the formation of new bone cells on the implant surface during the osseointegration process. Hence implant stability is associated with the quality and quantity of local bone [25]. Nowadays, the technique most frequently used to detect implant stability during healing times and in subsequent follow-ups is the non invasive diagnostic tool known as resonance frequency analysis (RFA) [26,27]. Continuous monitoring at various time points is important to determine the implant stability status and to estimate a long-term prognosis for successful therapy [28]. Evaluating bone density has long since been one of the most important parameters to quantify bone quality as it is thought to be a major determinant of primary stability. In other words, primary implant stability is dependent on not only the thickness of the bone into which the implant is placed, but also on bone density. Therefore, all these factors should be taken into account when making the clinical decision to perform a one-stage or a two-stage procedure [29]. The level of bone density at the implant site could be of utmost importance as it is related with failure rates and primary stability. When it comes to evaluating primary implant stability in relation with bone density, the implant stability quotient (ISQ) and the resonance frequency analysis can be used [30]. The primary stability of a dental implant, absence of mobility at the osseous site after implant insertion, and the quality of the receptor bone site are highly correlated. Likewise, primary stability is also strictly correlated to the mechanical relationship between the implant surface and the recipient bone. This relationship can determine implant placement outcomes by avoiding micromovements on the interface [31].

Optimal outcomes in implant survival terms have been demonstrated for implants placed in the maxillary sinus filled with deproteinized bovine bone mineral, and this material can be considered a safe predictable graft material for sinus floor augmentation [32]. However, very little is known about the physico-chemical properties of used grafts and the impact on both bone density and early implant stability after they have been employed. The aim of this randomized split-mouth design was to compare the stability of dental implants placed after sinus floor elevation with two HAs during the first six months of healing by means of RFA, and to monitor how the physico-chemical properties affect the implant stability quotient (ISQ) at the placement and healing sites.

## 2. Results

### 2.1. Graft Implants Characterization

**Figure 1:**
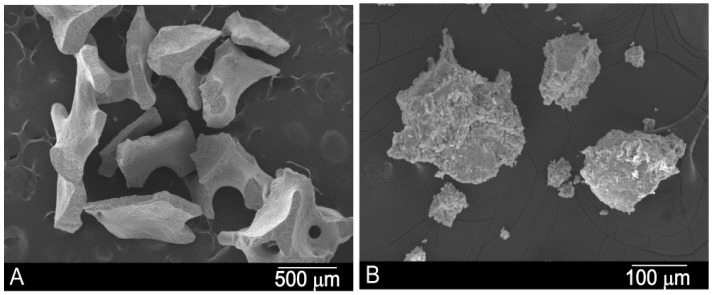
Scanning electron micrographs of (**A**) the BBM and (**B**) PBM xenograft materials before implantation.

Figure 1 shows the xenograft materials before inserting for maxillary sinus floor elevation. The BBM (bovine bone material) consists of HA particles of 500–1000 μm on average, with rounded edges and pores of 100 μm on average. The PBM (porcine bone material) consists of HA and collagen particles of 600–1000 µm on average. A summary of the microstructural parameter studied in a previous paper is shown in Table 1 to provide a better understanding of the materials´ microstructure [33].

### 2.2. Radiological and Thermographic Results

**Figure 2:**
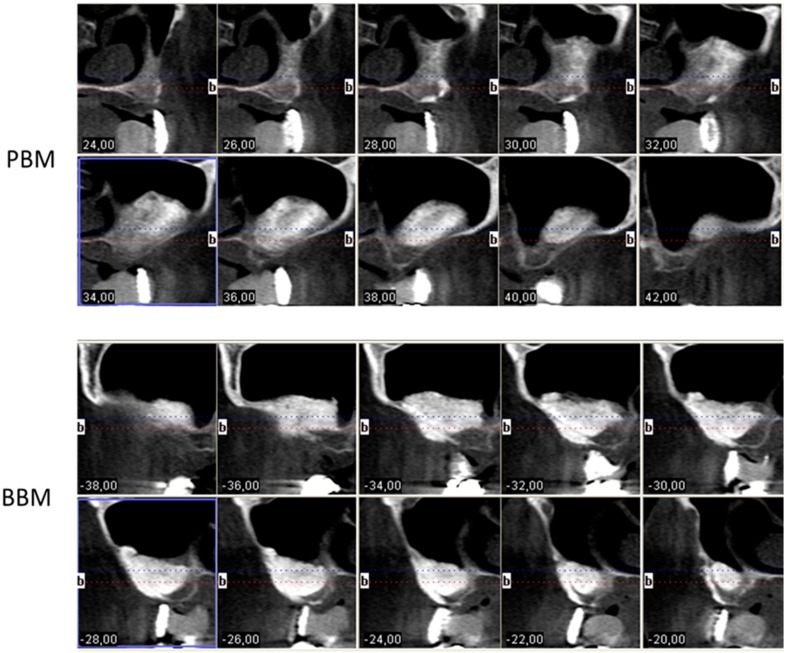
The i-CAT Vision postoperative image shows the increased volumes produced by the xenograft procedures 6 months after maxillary sinus elevation with a porcine hydroxyapatite (axial cuts 24–42) and a bovine hydroxyapatite (axial cuts −38 to −20)( b = Buccal side).

After a six-month follow-up period of our ten partially-edentulous patients treated with xenograft materials for sinus floor augmentation, the success rate was 100%. No sinus membrane perforation or other clinical complications, such as sinusitis or pain, resulted from surgery. The increased volumes produced by the xenograft procedures were stable by the end of the healing period, as seen in Figure 2. Deproteinized bone particles of two different temperatures induced osteoconduction six months after implantation.

**Figure 3:**
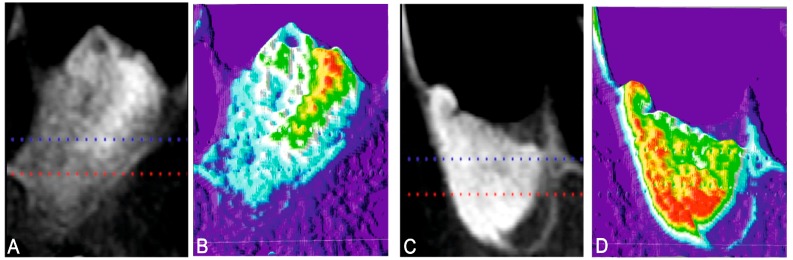
X-ray of the implant site six months after xenograft materials implantation and the corresponding color bone density (**A**,**B**) the PBM material and (**C**,**D**) the BPM material.

Bone density at the implant site could be crucial as it has been reported to correlates with failure rates and primary stability. The bone density in the grafted area with both biomaterials was evaluated radiologically as a routine diagnostic approach. Color thermal graduation was used to observe changes in radiopacity in the intrasinus bone grafted area. At the time of implant insertion, and after a six-month healing period, the augmentation sites treated with the PBM showed denser new bone formation, which was achieved along the inner surface of the replaced bony window than the area on the bone graft (Figure 3A,B). The BBM shows that denser new bone formation was achieved in the area on the bone graft compared to the original augmentation density (Figure 3C,D). The bone area differed in the groups after implantation and increased with time. Bone initially formed on the sinus wall and proliferated into the center of the augmented sinus cavity. Newly-formed bone came consistently into close contact with particles, and no gaps were present on the bone particle interface. Particles appeared to act as a scaffold by supporting new bone formation. Scaffolding is a critical component in tissue engineering because it provides the three-dimensional clues for cell seeding, migration and growth, and also for new tissue formation.

Although the radiopacity of the augmented volume increased with time for both xenograft materials, the receiving control sites showed a higher density of radiopacity compared with the PBM. Maxillary sinus membrane preservation is important to avoid the displacement of graft materials into the sinus cavity. However, nonobserved small perforations can imply a risk if left untreated.

### 2.3. ISQ Results

Primary implant stability in relation with bone density can also be evaluated by the implant stability quotient (ISQ). Detailed distributions for the implants and ISQ values over the investigated time periods are depicted in Table 2. Three implants were not osseointegrated at the end of the study, which left 57 implants for controls (a 95% success rate). Dropouts were not observed during the evaluation period. 

**Figure 4:**
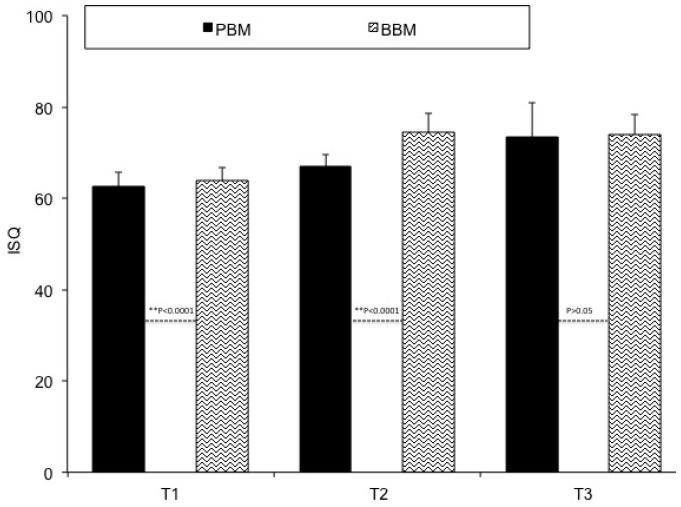
Implant stability quotient for both xenograft materials.

The ISQ (Baseline) averaged values were 63.8 ± 2.97 for a sintered BBM and 62.6 ± 2.11 for a non-sintered PBM, and differences were statistically significant. The ISQ (Stage 2) average values were 73.5 ± 4.21 for the BBM and 67 ± 4.99 for the PBM, and differences were statistically significant. The ISQ (Stage 3) average values were 74.65 ± 2.93 for the BBM and 72.9 ± 2.63 for the PBM, and differences were statistically significant. The detailed distributions for the groups are depicted in Figure 4. The analysis of variance demonstrated a statistically significant difference (*p* < 0.0001).

In the vestibule-lingual direction, the mean and standard deviation ISQ values measured at Baseline, Stage 2, and Stage 3 were 63.4 ± 2.88, 73.9 ± 4.11, and 73.8 ± 2.99, respectively, for the BBM, and were, respectively, 62.4 ± 2.92, 66.9 ± 2.67, and 72.6 ± 7.67 for the measured PBM ISQ values.

In the mesio-distal direction, the mean and standard deviation ISQ values measured at Baseline, Stage 2, and Stage 3 were 64.2 ± 3.18, 75.2 ± 4.29, and 74.2 ± 3.01, respectively, for the BBM, and were, respectively, 62.8 ± 3.23, 67.1 ± 2.33, and 74.2 ± 7.59 for the measured PBM ISQ values.

The multivariate regression analysis (R2 adjusted = 0.58, multiple correlation coefficient = 0.78) demonstrated significant influences on the ISQ values in relation to age (*p* = 0.0120; *r* = −0.13), gender (*p* < 0.0001; *r* = −0.48), group (*p* = 0.0017; *r* = 0.16), position (*p* = 0.0002; *r* = 0.19), and time (*p* < 0.0001; *r* = 0.69).

## 3. Discussion

This clinical study describes a comparison of the RFA of implants placed and delayed with maxillary sinus grafting with two biomaterials deproteinized at different temperatures during three distinct time periods. Sixty implants were fitted in 10 patients. Three implants were lost throughout the study time period, and the survival rate of dental implants in the present study was 95%. The results also showed a positive correlation between the used biomaterial and the ISQ values. The results of a systematic review of the survival of implants in bone grafts, Aghaloo and Moy, found that the survival of implants by the maxillary sinus grafting technique was 95.6% [2]. Presently, no agreement about the advantages of using grafting material in maxillary sinus elevation techniques with dental implant insertions has been reached because the question whether these techniques and materials may determine implant survival, compared to pristine bone, remains unsolved [34,35]. To find the answer to this question, long-term stability up to 20.2 years was retrospectively examined after the placement of implants at both augmented and non augmented sites. The results of this retrospective study determined that the implants inserted into an augmented site had a similar implant survival to those inserted into non augmented sites [36]. Based on evidence, it can be stated that the implants inserted into augmented bone offer a similar implant survival to those placed in native bone [37]. In our study the implicated grafting material might not compromise implant survival.

For clinicians, one of the most important parameters to measure the scope of mechanical loading capability is implant stability. The baseline information that it provides serves as a tool to evaluate clinical outcomes and time courses [28]. Several attempts have been made to find innovative techniques that allow implant stability to be measured [27]. An RFA, which is a non-invasive technique that presents highly reproducible results, has been proposed [23,38,39]. This technique has become one of the most widely used techniques to measure implant stability immediately, which makes determining the probable loading protocol and assessing the long-term survival of implants possible [25]. The comparison of the type of bone graft and initial implant stability was performed herein. The results of this study showed that the implants placed and delayed (two-stage) with the maxillary sinus lift with different biomaterials presented distinct ISQs, with statistically significant differences. 

From a clinical point of view, it would appear relevant to know the significance of RFA measurements and the relationship between their values and implant osseointegration success or failure. A previous study has observed that implants retrieved after 6 months show a strict correlation between the RFA values and the percentage of bone implant contact (BIC) [40]. The aim of the present study was to determine whether the same correlation existed at earlier time points, specifically in implants inserted after three or six months. A statistically significant correlation was detected between the RFA values and healing time. During the bone-healing period, the implants’ ISQ value varied with time. In the surgical phase (baseline), the average ISQs for all the implants were 63.8 ± 2.97 for a sintered BBM, and 62.6 ± 2.11 for a non sintered (PBM), with statistically significant differences. The ISQ (Stage 2) average values were 73.5 ± 4.21 for the BBM and 67 ± 4.99 for the PBM, where differences were statistically significant. The ISQ (Stage 3) average values were 74.65 ± 2.93 for the BBM and 72.9 ± 2.63 for the PBM, with statistically significant differences.

The present study examined the same implants three and six months after their installation for the implants inserted by a two-step procedure. The RFA values after three months showed that the grafted sites provided good implant stability and, on average, the BBM were better than the PBM group sites. This difference was still present after 6 months and was also statistically significant. The importance of bony quality in relation to implant stability has been previously highlighted. Given the relative lack of bone, there is some concern about the initial stability of the implants placed in grafted bone. An RFA enables the stability under load to be qualitatively measured, and has been advocated as a means of assessing implant stability at the time of placement and in later phases when providing restorations [41]. 

This is the first study to compare ISQs with density values after sinus lift procedures six months after healing and primary stability. The primary initial stability (IS) is a crucial factor to establish osseointegration [42,43,44], and might be subject to the influence of the following factors: bone quality, surgery technique, and implant macrodesign [44].

The above studies have demonstrated a strong correlation between implant displacement and bone properties, and have concluded that better bone quality leads to better implant stability. Al-Khaldi has also proven that implants with a high degree of density placed in bone have higher initial stability values than those placed in soft bone [45], according to the results of insertion torque (IT), ISQs and removal torque values (RTV) [46]. However, ISQs can improve through changes in the implant macrodesign [47]. Moreover, when using a different drilling protocol in soft bone, Sennerby et al. confirmed that when comparing tapered implants with parallel implants, the former showed a higher primary stability than the latter [48].

A previous study on the same pool of implants used in our study (tapered implants) demonstrated that grafted bone can offer good primary stability to implants and that, during the surgical procedure, only a few mechanical characteristics of implants (length and diameter) were able to influence ISQ values [49]. Degidi et al. have suggested that the length and width of implants can influence primary stability because of the increased bone-implant contact surface area [50]. In the present study, the macrodesign, and the length and diameter of the implant were not used as evaluation factors as all the used implants were 4 mm in diameter and 11.5 mm in length, with tapered internal hexagon implants, and are consistent with these studies, which report no statistically significant differences in ISQ due to ISQ length or diameter [51,52]. From the clinical point of view, implants that have been placed in soft or grafted bone show better mechanical stability values when they have a narrow diameter and a tapered macrodesign [53,54]. In fact nearly every implant company offers tapered-design implants for alveolar ridges with deficient bone quality and quantity.

This should be taken into account for alveolar ridges with deficiencies in bone quality and quantity, which is the reason why almost every company in the sector has introduced tapered-design implants.

In our study we used a small particle size of two different deproteinized bone grafts. Jensen et al. evaluated the influence of the particle size of DBBM on bone formation and implant stability when used for sinus floor elevation in a mini-pig model. In the initial healing phase, small particle size DBBM showed marginally higher osteoconductive capacity than large particle size DBBM. However, no differences were observed in the amount and speed of bone formation, BIC or implant stability between the two test groups. However at the baseline and at six and 12 weeks, the BIC values were comparable, or even higher, than in our study in humans. Primary implant stability is dependent on not only the thickness of the bone into which the implant is placed, but also on bone density, thread configuration, and implant shape, and the presence of an implant neck. Therefore, all these factors should be taken into account when making the clinical decision to perform a one-stage or a two-stage procedure [55]. 

As evidenced in the review of Browaeys et al. on using biomaterials in sinus augmentation, within the limitation of the animal studies examined, and based only on histological examinations, the biomaterial used in the grafting procedure does not influence the initial osseointegration of dental implants [56]. 

The correlation between the RFA values and good bone quality reported herein seemed to confirm the different importances of the factors that determine RFA values upon implant insertion and if it is able to maintain this stability after three or six months. In fact, good quality bone probably reacts better to implant insertion, and implant stability after bone remodeling could be greater.

Long-term stability has also been reported by Hallman et al. In their study 108 dental implants were placed six months after sinus floor augmentation with a mixture of autogenous and deproteinized bovine bone. After three years of loading, implant stability was recorded using an Osstell instrument. The mean reported RFA values were 67.4 ± 14.5 for residual bone and 65.6 ± 13.8 for the augmented sites. Unfortunately, no more data are currently available about the importance of bone quality in determining long-term RFA values, so more studies have to be conducted [57].

Healing times of 6–9 months before implant placement are usually recommended for sinus elevation in combination with grafting material [58], and an additional 3–6-month period of implant healing time is needed. However, extended integration periods and multiple surgeries pose a challenge for patient acceptance [59]. 

Our results also indicate that regardless of the substitute material, the implants inserted into a grafted sinus can be predictably loaded as the implants inserted into a grafted area. Previous studies have concluded that the prognosis of implants inserted into augmented sinuses and the fixed restoration supported by these implants do not appear to be influenced by factors, such as graft material, restoration type, residual bone height, and time of implant placement. Within the limits of this review, the prognosis of implants and fixed restorations did not seem to be influenced by the restoration type, graft material, residual bone height, and time of implant placement. However, the conclusions of this review are based on studies that provide a low level of evidence. Therefore, careful interpretations are required. Multicenter randomized controlled clinical trials with sufficient statistical power that concentrate on a few factors are needed to draw sound conclusions [11]. 

The differences between the two HAs, in porosity, crystallinity, density, surface area and composition terms, may determine different behaviors of this material, and might affect early implant stability in this clinical situation. The HA of a bovine origin sintered with high crystallinity, low porosity, high density, and a larger granule size presents better stability, which demonstrates that variations in the physico-chemical properties of a bone substitute material clearly influence implant stability. A profound knowledge of the graft material characteristics is of utmost importance when assessing their clinical outcomes. The physico-chemical properties of bone substitute materials can influence osseointegration and shorten healing times from implant placement to restoration. Understanding not only the biomaterial properties, but also their relation and influence on bone healing, seems crucial.

## 4. Materials and Methods

### 4.1. Commercial Xenograft Materials

The commercial graft materials used in this study were two different types of bone deprotenized hydroxyapatite materials of diferent origins employed in dentistry: deproteinized porcine bone mineral hydroxyapatite (PBM), called OsteoBiol^®^ (OsteoBiol, Tecnoss Dental SRL, Torino, Italy); deproteinized bovine hydroxyapatite (BBM), called Endobon^®^ (RegenerOss™, BIOMET3i, Palm Beach, FL, USA). The physico-chemical and morphological characterizations of both xenograft materials are found in a previous study [33].

### 4.2. Implant Procedure

#### 4.2.1. Patient Selection and Protocol

Ten partially-edentulous patients (five females and five males), whose ages ranged from 37 to 60 years, attended the Department of Oral and Maxillofacial Surgery. Patients who demanded fixed restorative appliances in the posterior maxilla were selected for maxillary sinus augmentation because sufficient bone tissue was lacking to place endosseous dental implants. The protocol for harvesting bone samples was approved by the University Ethics Committee and informed consent was obtained from all the patients (UCAM-Ethics Committee, approval ID: 6637). The study was designed following the Declaration of Helsinki guidelines for experimentation on human subjects. Any possible complications that could arise from surgical therapy were treated following standard dental management protocols.

#### 4.2.2. Inclusion and Exclusion Criteria

Atrophy of the lateral-posterior maxilla and residual crestal bone height was classified according to Cawood et al.’s subsummizing classes I–VI. All of the patients underwent CBCT before surgery as a routine diagnostic approach to carefully evaluate the available bone at the intended surgical site and for planning the grafting procedure. 

The inclusion criteria were as follows: maxillary partial bilateral edentulism that involves premolar-molar areas. The cases with a crestal bone height between 7 mm and 0 mm, and with high postero-lateral atrophy (Cawood V–VI), are most likely to undergo a two-stage lateral antrostomy. 

The exclusion criteria were: patients who suffer from an uncontrolled systemic disease or a condition known to alter bone metabolism (i.e., osteoporosis, diabetes mellitus, etc.); subjects who were taking/had taken medications known to modify bone metabolism; e.g., bisphosphonates, corticosteroids, etc.; women who were pregnant or trying to get pregnant at the time of screening; patients who presented existing sinus conditions, sepsis, a history of cancer and/or radiation to the oral cavity; or complications derived from any of these conditions that affect the sinus area.

#### 4.2.3. Surgical Procedure. First Phase

**Figure 5:**
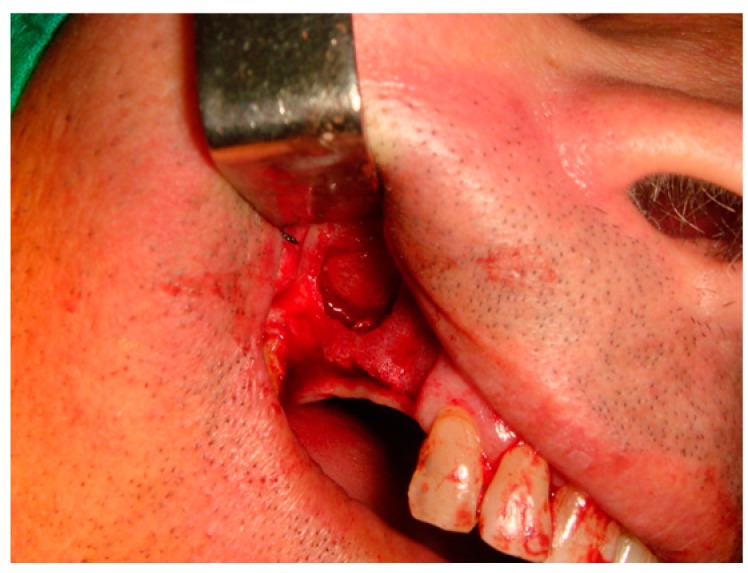
A lateral window is prepared and the Schneiderian membrane is elevated.

The study was performed in two surgical phases. In the first phase all the patients took 875/125 mg of amoxycillin/clavulanic acid every 8 h starting one day before surgery. A 300-mg dose of clindamycin every 8 h was prescribed to penicillin-allergic patients. This medication was maintained for seven days. All of the surgical procedures were performed under local anesthesia (Ultracain, Aventis Inc., Frankfurt, Germany). The basic surgical procedure was represented in all the patients by maxillary sinus floor elevation via a lateral approach, as described by Boyne and James (Figure 5). 

**Figure 6:**
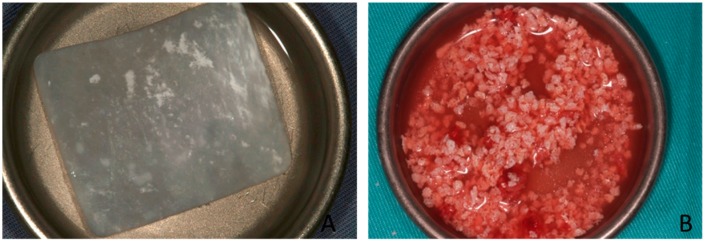
(**A**) A short-term absorbable collagen membrane; (**B**) Grafting materials mixed with venous blood.

After membrane elevation, a bioabsorbable collagen barrier membrane was placed under the sinus membrane and adapted to come into contact with peripheral bony walls (Evolution Fine, OsteoBiol^®^, Tecnoss Dental S.R.L., Torino, Italy). Sinuses were allocated to the non-sintered HAs (PBM) or to the sintered HAs (BBM) group via a standard randomization protocol (http://www.randomization.com). On one side, sinus cavities were grafted with the PBM (OsteoBiol^®^ mp3, Tecnoss Dental S.R.L., Torino, Italy). After grafting, an absorbable collagen membrane (Evolution Fine^®^, OsteoBiol^®^, Tecnoss Dental S.R.L., Torino, Italy) was placed over the window to minimize soft tissue invasion. On the other side, sinus cavities were grafted with the BBM (Endobon^®^, RegenerOss™, BIOMET3i, Palm Beach Gardens, FL, USA). The grafting materials were mixed with venous blood from the defect area and were carefully packed in the created volume following mucous membrane elevation. After bone grafting, a short-term absorbable collagen membrane (Evolution Fine, OsteoBiol^®^, Tecnoss Dental S.R.L., Torino, Italy) was placed over the window. Primary closure was achieved in both cases by suturing with 3–0 silk suture (Laboratory Aragón, Barcelona, Spain). Sutures were removed two weeks after surgery. During the postoperative period, patients were clinically and radiologically followed up at monthly intervals (Figure 6).

#### 4.2.4. Radiographic Thermal Imaging Analysis

**Figure 7:**
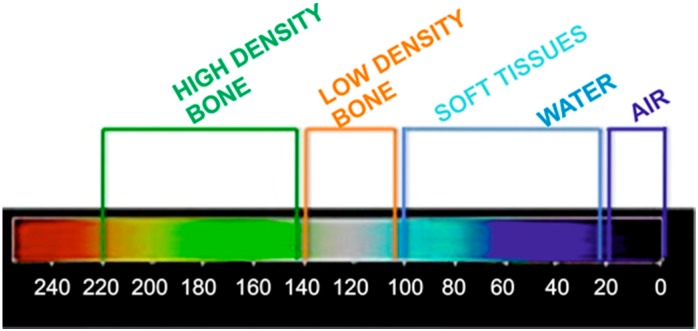
Color-graduated density scale for thermal imaging interpretations.

During the post-operative period, patients were clinically and radiologically followed up at monthly intervals. All the patients underwent CBCT six months after surgery as a routine diagnostic approach using an I-CAT^®^ (Imaging Sciences International, Hatfield, PA, USA) Cone Beam 3D and were analyzed using the I-Cat vision software. The obtained images were processed by Image J software (Bethesda, MD, USA), developed by the National Institute of Health (NIH) of the USA; a 3D plug-in with a thermal LUT and a grid size of 128 × 128, smoothing of 6.0, and a perspective of 0.2 on a 1:1 scale. Color thermal graduation was used to observe the changes in radiopacity in the grafted area (intrasinus bone graft). The scale was graduated with values from 0 to 240, where 0–20 were the values assigned to air, 20–100 were the values assigned to water and soft tissues, 100–140 were the values assigned to lower density bone, and 140–220 were the values assigned to higher density bone (Figure 7). 

#### 4.2.5. Surgical Procedure: Second Phase—Implant Insertion

The second surgical phase was performed after the healing period. Functional implants were placed on each side. Each side received three implants (3i T3^®^, Certain Tapered Internal Connection Implants, BIOMET 3i™, Palm Beach Gardens, FL, USA) placed six months after augmentation. All of the patients took 875 mg/125 mg units of amoxycillin/clavulanic acid every 8 h starting one day before surgery. A dose of 300 mg of clindamycin every 8 h was prescribed to penicillin-allergic patients. This medication was maintained for seven days. All of the surgical procedures were performed under local anesthesia (Ultracain, Aventis Inc., Frankfurt, Germany) in an outpatient setting by the same surgeon, who was familiar with the implant system. For the procedure, a full thickness mucoperiosteal flap was elevated on the sides. The osteotomy using a conical drill, with copious irrigation using saline solution, was done on the crest of the bone. Then osteotomies for fitting implants were produced using the initial drill to determine the depth and direction of the site. Afterward, implants were positioned in the local area, predetermined at the crestal bone level. Sixty conical implants with an internal hexagon connection were applied, which were all 11.5 mm long with a 4.0 mm diameter. Implants were selected according to the prior evaluation of each case. All the implants were fitted using surgical guides, and wounds were sutured in a tension-free state. Antibiotics and analgesics were given for one week. All of the patients took 875/125 mg of amoxycillin/clavulanic acid every 8 h starting one day before surgery, and 300 mg of Clindamycin every 8 h werre prescribed to penicillin-allergic patients. This medication was maintained for seven days. Patients were asked to rinse with 0.12% chlorhexidine three times daily for two postoperative weeks. Sutures were removed two weeks after surgery. All of the implants were prepared with a healing abutment until rehabilitation commenced.

### 4.3. Measuring Implant Stability

**Figure 8:**
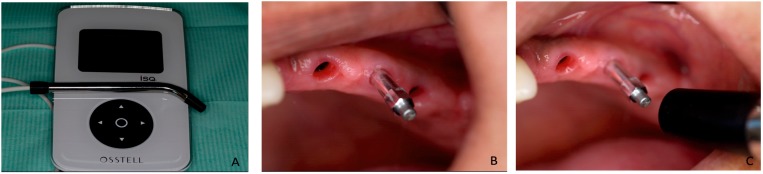
(**A**) Implant-bone contact rigidity was measured by RFA (Osstell™ Mentor, Integration Diagnostic AB, Sweden). RFA measurements were obtained before the healing cape was screwed into implant fixtures; (**B**) A Smartpeg (Smartpeg™, Integration Diagnostic AB, type 4 regular neck) was attached manually to the fixture with the help of a mount, and a torque of 4–5 Ncm was applied; (**C**) All the measurements were taken out by the same researcher. Measurements were taken at the time of implant placement and (baseline) at Stages 2 and 3. In all cases, an ISQ was calculated as the average of four measurements per implant (twice in the bucco-lingual direction and twice in the mesio-distal direction).

After dental implant insertion, the resonance frequency evaluation was made using the Ostell™ Mentor (Integration Diagnostics AB, Göteborg, Sweden) to measure the implant’s primary stability. A Smartpeg™ (Integration Diagnostics AB, Göteborg, Sweden) was placed in each implant and was tightened to approximately 4–5 Ncm. The transducer probe was aimed at the small magnet at the top of the Smartpeg and at a distance of 2–3 mm, and was held stable during pulsing until the instrument beeped and displayed the ISQ value (Figure 8). The RF value is represented by a quantitative parameter called ISQ. The ISQ range went from 1 to 100. An increased ISQ indicates increased stability, whereas low values indicate reduced implant stability. The ISQ values were measured during the surgical procedure (T1-baseline), at three months (T2) after surgery, and at six months (T3) after surgery. Measurements were taken twice in the bucco-lingual direction and twice in the mesio-distal direction. The mean of the two measurements in each direction was regarded as the representative ISQ for that direction. The higher values were recorded for the bucco-lingual (B-L) direction and the mesio-distal (M-D) direction. The ISQ values were separately evaluated. Each implant was also evaluated during all visits for mobility, pain and signs of infection.

### 4.4. Statistical Evaluation

All of the data were recorded, reviewed, and inputted into a computing system. Analyses were performed using specific software (MedCalc v15.8, Ostend, Belgium). The influence of age, gender (male, female), group (BBM, PBM), ISQ measurement direction (BL, MD), and the evaluation time period, (T1) baseline, (T2) three months, abnd (T3) six months, on the ISQ values was evaluated by an analysis of variance (ANOVA) and multiple regression at the 5% significance level (backward method) with the help of appropriate software (MedCalc v15.8, Ostend, Belgium).

## 5. Conclusions

The differences between the two HAs found in porosity, crystallinity, density, surface area, and composition terms may determine the different behaviors of this material and might affect early implant stability in this clinical situation. The HA of a bovine origin (BBM) sintered with high crystallinity and low porosity presents better stability, which demonstrates that variations in the physico-chemical properties of a bone substitute material clearly influence implant stability. Detailed information about the graft material’s characteristics is crucial to evaluate its clinical outcomes. The influence of the physico-chemical properties of bone graft materials on osseointegration has led to shorter healing times from implant placement to restoration. A sound understanding of various aspects of biomaterial properties, and their relation to and influence on bone healing, is of utmost importance.

## Figures and Tables

**Table 1:** Physical properties of the two xenograft materials [33].

Material	Phase/s	Ca/P Ratio	Particle Size (μm)	Crystal Size (nm)	Real Density (g/cc)	Porosity (%)	Surface Area (m^2^/g)
**PBM**	HA+Coll	2.22 ± 0.08	600–1000	325	2.85	59.90	97.84
**BBM**	HA	2.31 ± 0.09	500–1000	732	2.98	49.13	2.77

**Table 2:** Demographic data.

**Number of Patients (Total)**	**10**					
**Number of Implants (Total)**	**60**					
***Osseointegrated (%)***	57 (95)					
***Non Osseointegrated***	3 (5)					
**Vestibule-Lingual (ISQ Values)**	**T1**		**T2**		**T3**	
	**PBM**	**BBM**	**PBM**	**BBM**	**PBM**	**BBM**
***Mean***	62.4	63.4	66.9	73.9	72.6	73.8
***SD***	2.92	2.88	2.67	4.11	7.67	2.99
***Median***	62.5	63.2	66.6	70.6	73.7	74
**Mesio-Distal (ISQ Values)**	**T1**		**T2**		**T3**	
	**PBM**	**BBM**	**PBM**	**BBM**	**PBM**	**BBM**
***Mean***	62.8	64.2	67.1	75.2	74.2	74.2
***SD***	3.23	3.18	2.33	4.29	7.59	3.01
***Median***	62.5	64.8	69.1	72.4	75.8	75.5

ISQ: Implant stability quotient; T1: baseline; T2: at three months; T3: at six months, and the means and standards deviation of the ISQ values.
